# Driving Innovation: Transatlantic Attitudes to the *Bionics Bus* as a Vehicle for Health Transformation and STEM Engagement

**DOI:** 10.1049/htl2.70090

**Published:** 2026-06-18

**Authors:** Christopher James, Sarang Shankar, Madeleine Dale, Bhathika Perera, Richard Laugharne, Matthew Allen, Maria Feijoo‐renones, Rohit Shankar

**Affiliations:** ^1^ Biomedical Engineering Institute University of Warwick Warwick UK; ^2^ Truro School Truro UK; ^3^ University Hospitals Plymouth NHS Trust Plymouth UK; ^4^ University College London UK; ^5^ CIDER University of Plymouth Peninsula School of Medicine Truro UK; ^6^ Department of Intellectual Disability Neuropsychiatry Research Team, Cornwall Partnership NHS Foundation Trust Truro UK

**Keywords:** assisted living, biocybernetics, biomedical measurement, blind source separation, brain, electroencephalography, medical signal processing, neural nets, patient monitoring, time‐frequency analysis

## Abstract

Emerging mobile health and STEM engagement platforms are increasingly recognised as catalysts for improving public understanding of biomedical technologies and supporting digitally enabled models of healthcare delivery. The *Bionics Bus* (https://bionicsbus.org/) is a novel mobile laboratory designed to democratise access to robotics, artificial intelligence, prosthetics and digital diagnostics by delivering hands‐on experiences directly to schools, communities and healthcare settings. This study provides the first transatlantic comparison of public attitudes toward the *Bionics Bus* within the United Kingdom and the United States of America (USA), two countries with contrasting healthcare systems and digital transformation priorities. Using a mixed‐methods cross‐sectional design, 78 participants completed a survey assessing technology perceptions, healthcare accessibility, enthusiasm for STEM and views on the usefulness and potential of the *Bionics Bus*. Quantitative analysis demonstrated high levels of excitement and strong recognition of the relevance of technology in healthcare across both countries, with USA respondents more likely to report geographical barriers to accessing healthcare. Thematic analysis revealed shared optimism for the *Bus* as a tool for education, accessibility and community‐based innovation. These findings highlight the potential of mobile biomedical platforms to support digital inclusivity, strengthen science capital and enhance public engagement with technology‐enabled healthcare.

## Graphical abstract

This study explores UK and USA participants views on using mobile platforms such as the *Bionics Bus* to support digital inclusion across communities. The *Bionics Bus* is seen as a tool to enhance STEM education and engagement. The public shows a strong enthusiasm for the mobile *Bionics Bus* concept.

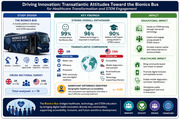

## 1 Introduction

Science, technology, engineering and mathematics (STEM) education is increasingly recognised as a critical driver of healthcare innovation, economic resilience and societal wellbeing in the 21st century [1]. Global health systems face escalating pressures from ageing populations, workforce shortages and chronic disease, all of which demand technologically enabled and scientifically literate responses [2]. Digital transformation is now a central pillar of policy in the USA and the UK, with artificial intelligence (AI), robotics and biomedical technology expected to redefine clinical decision‐making, prevention and personalised care [3, 4]. Yet, despite this momentum, gaps persist in public understanding, digital literacy and equitable access to STEM education, which risk widening socioeconomic and health inequalities [5, 6]. The World Health Organisation and the Organisation for Economic Co‐operation and Development have emphasised that empowering communities with STEM knowledge and digital skills is essential for achieving sustainable, inclusive health innovation [7, 8]. Cultivating STEM engagement, therefore, represents not only an educational priority but a public health imperative, underpinning equitable participation in the technological future of healthcare [9].

### 1.1 Policy Context: The United Kingdom (UK)

The recent white paper from the government outlines reforms to the post‐16 education and skills system in England, aiming to meet the needs of the economy, close skills gaps and support growth. It emphasises the importance of aligning education with emerging technologies and the evolving needs of the healthcare sector [10].

The UK's health system is entering a decisive period of transformation driven by the need to align healthcare delivery with emerging technologies, community‐based care and prevention‐led health strategies [11]. The *Fit for the Future: 10‐Year Health Plan for England* outlines an ambitious reform agenda for the National Health Service (NHS), centred around three radical shifts: moving from *hospital to community*, from *analogue to digital* and from *sickness to prevention* [11]. These reforms, often referred to as the ‘Darzi Shifts,’ reflect a structural reimagining of the NHS in the face of demographic pressures, health inequalities and technological opportunity [11]. The first shift from hospital to community reorients care delivery toward neighbourhood‐based services and decentralised health infrastructure [11]. The second shift from analogue to digital aims to make the NHS ‘the most AI‐enabled health system in the world,’ integrating innovations in data, robotics, genomics and digital diagnostics [11]. Achieving this requires both technological investment and societal readiness, public trust, understanding and workforce capability [12]. The third shift from secondary and tertiary prevention to primary prevention prioritises proactive health management through early education, self‐efficacy and community participation [11].

### 1.2 Policy Context: The United States of America (USA)

The USA has implemented a multifaceted approach to enhance digital literacy within its STEM education framework [13, 14, 15]. The plans emphasise the integration of digital literacy across all educational levels, aiming to equip students with the necessary skills to navigate and contribute to a technology‐driven society [13, 14, 15]. Key objectives include fostering computational thinking, promoting digital fluency and ensuring equitable access to STEM opportunities for all learners. This includes engaging students from diverse backgrounds in STEM education, thereby addressing disparities and promoting digital literacy as a fundamental component of modern education [14]. Another strategy underscores the importance of digital literacy in fostering innovation and maintaining global competitiveness [15]. Collectively, these policies reflect a concerted effort by the USA government to integrate digital literacy into the fabric of STEM education, ensuring that students are well‐prepared to meet the challenges of an increasingly digital world. This is broadly in keeping with the UK government policy.

By contrast, healthcare policy and innovation are embedded in a markedly different institutional and ideological context. The Centres for Medicare and Medicaid Services and the Office of the National Coordinator for Health have launched national strategies that prioritise interoperability, digital infrastructure and patient empowerment over universal service [16, 17]. Meanwhile, initiatives such as the National Association of Community Health Centres’ mobile health programme have expanded mobile outreach through private and philanthropic partnerships [18]. These strategies emphasise patient choice, data‐driven care and technology‐enabled convenience within a market‐based healthcare ecosystem.

### 1.3 The *Bionics Bus*

The term ‘bionics’ was coined by Jack E. Steele (a medical doctor and American Air Force Colonel and worker at the Aeronautics Division House at the Wright‐Patterson Air Force Base in Dayton, Ohio) in 1958 [19]. We adopt the term bionics here, although initially it represented biologically inspired engineering, which is the application of biological methods and systems found in nature to the study and design of engineering systems and modern technology. Bionics now has a wider meaning and deals with the technical implementation and application of constructional, processing and developmental principles of biological systems.

The *Bionics Bus* (https://bionicsbus.org/) is an innovative mobile STEM engagement and education platform designed to democratise access to biomedical and digital health innovation [19]. Developed via collaborations between academic institutions, the NHS, local communities and industry partners, the *Bus* is a mobile laboratory that brings hands‐on experiences in robotics, prosthetics, artificial intelligence and wearable health devices directly to schools, community centres and healthcare sites [18]. By integrating educational outreach with public health awareness, the *Bus* addresses key objectives of stimulating interest in STEM careers, supporting workforce development and fostering ′science capital among underrepresented groups [18].

### 1.4 The Applicability of the *Bionics Bus*

Empirical work on the *Bus* shows strong support in the UK context. The project's first study was done in 2024, inquiring into the attitudes of *Bionics Bus* concept of the general public [19]. In the mixed‐methods study (*n* = 54), 96% of participants expressed excitement about the concept and 94% agreed that technology should play a greater role in healthcare. Qualitative themes that emerged included accessibility, technology as progress and inspiration through hands‐on experience. These responses reflect to some extent the UK's public‐service ethos, where technology is framed as a collective good that promotes inclusion and health equity.

It can be argued that the *Bionics Bus* directly supports the NHS's three core policy shifts [11]. It extends innovation into communities, familiarises citizens with AI and robotics and fosters early engagement in health and science [11]. It acts as a bridge between academia, clinical practice and community engagement, embodying the regional innovation model [20, 21].

In the USA context, the *Bus* would require adaptation to align with a more individualised and market‐driven model of innovation. American attitudes toward bionics often centre on autonomy, personal empowerment and technological frontierism, rather than collective wellbeing [22]. Comparative research has shown that UK participants emphasised trust and transparency [23]. These distinctions highlight that while both countries are optimistic about technology, societal framing differs in theory. In the UK, bionics are seen as tools for equity and prevention, while in the USA as pathways to performance and personalisation.

Understanding these transatlantic differences is essential to designing culturally resonant public engagement models [24]. Comparing transatlantic attitudes is important for mutual learning, improving policy and fostering innovation across different health and science systems [25]. Understanding these differences helps policymakers and health professionals address common challenges and tailor effective solutions to their specific populations. The *Bionics Bus* can offer a comparative testbed for studying how healthcare systems, culture and policy shape public trust in biomedical technologies.

## 2 Aim

Comparing attitudes toward the *Bionics Bus* (https://bionicsbus.org/) and biomedical technologies between the USA and the UK. *Study design and participants*

The study followed the (strengthening the reporting of observational studies in epidemiology) STROBE checklist for reporting the cross‐sectional study. The STROBE checklist consists of 22 essential items. It is designed to ensure transparent and comprehensive reporting of observational studies. It was conducted at the STEM Workshop on board the USS Hornet, in San Francisco, as part of ISTAS25 on the seventh of September 2025. We used the data collected at the Annual Classic Motor Show at the National Exhibition Centre, Birmingham, UK, held from 8–10 November 2024, which has been published to validate the concept, to provide a UK comparator group [19]. We defined the elements of our study design into a quantitative approach of identifying elements of *technology and healthcare*, *accessibility of healthcare systems* and *usefulness of Bionics Bus*. For the qualitative part, we looked into categories of *relevance of technology and healthcare*, *usefulness of Bionics Bus* and *potential of Bionics Bus*.

### 2.1 Study Material

The study material was a stall in the exhibition where knowledge of the bus, including its description and its purpose, was put on a large screen and in a continuous loop for people to engage with. Alongside it were handouts and poster material outlining key aspects of the bus technology. Along with this, there were active and regular demonstrations of the different pieces of technology hosted by the bus. This included smart watches and their utility in capturing blood pressure, heart rate and so on, mobile and wireless EEGs informing people on their brain activity and so on.

### 2.2 Survey Tool

A brief online survey was developed by the study team to obtain the views of the general public (Supporting Information S1). The survey was designed so that specific respondents would not be providing personal information that would render them identifiable. Explicit informed consent to pool data and publish was obtained. Attendees were provided with information and video demonstrations relating to the functionality of the *Bionics Bus*, with a prototype of the Bus also on display (in the UK, in the USA, the *Bionics Bus* concept was described and illustrated on video alone). Question items related to the demographic characteristics of the respondents (e.g., age group, ethnicity, citizenship, region of residence, level of education, current health concerns, familiarity with technology, access to healthcare and healthcare technology, thoughts on the *Bionics Bus*), their views on implementation to increase healthcare technology driven accessibility particularly on neurological and neuropsychiatric conditions was captured using 17 Likert questions with 5 responses and 3 open questions. This included barriers that they perceived the Bus would encounter.

### 2.3 Ethics

This study is primarily a public consultation to gain insight into how a novel approach should be communicated to the public and delivered in a way which provides an acceptable, understandable, optimal user experience, which is inclusive and ethical [26]. It is focused on engaging *with* different communities *by* the survey to inform on the *Bionics Bus* utility [26].

All participants were given a live demonstration of the bus and a description of the proposed tools and concepts. Post this, they were asked if they would like to participate in this study to give their views on how to engage and improve populations in their communities using the *Bionics Bus*. Those who gave informed consent to do so were advised at the start of the study that participation was voluntary and their replies, if they chose to participate, would be anonymised and analysed. Completion of the survey was regarded as consent to participate in the study and was explicitly mentioned to each participant as such. Separate informed consent (or refusal) was taken from all participants to use their submitted data for publication. No participant identifier data was collected. Contact details (email) of the first author were provided to participants to provide feedback, any concerns, thoughts or requests to withdraw their data collected. Data were pooled prior to analysis.

### 2.4 Statistical Analysis

The survey employed convenience sampling using face‐to‐face discussion and providing a link to an online form for people to complete in their own time on their mobile/computer. The survey link was open for the duration of the Show.

Quantitative survey data were analysed descriptively and compared between sites (UK and USA). Categorical variables were summarised as frequencies and percentages. Differences in proportions between the two sites were assessed using Fisher's exact tests. This approach was applied to all categorical variables, including participant demographics (sex, age group, ethnicity, nationality, education level) and survey responses relating to technology attitudes, healthcare accessibility and perceived usefulness of the *Bionics Bus*. For multi‐option questions where participants could select more than one response (e.g., self‐reported disabilities), each category was treated as an independent binary variable and analysed separately using Fisher's exact test. Data were analysed using R (Version 4.4.0). Significance was taken at *p* < 0.05.

### 2.5 Qualitative Analysis

Qualitative data from the four open questions (usefulness, relevance, potential and proposed contents) in the USA and UK surveys were thematically analysed following Braun and Clarke's six‐phase approach [27]. This included (1) familiarisation with data collected on the *Bionics Bus* project, (2) systematically identifying meaningful features from the comments provided by the participants, (3) grouping the comments into themes, (4) reviewing themes for coherence within and distinction between themes, (5) defining and naming themes and (6) putting them together as a coherent narrative.

## 3 Results

There were 82 respondents (54 from the UK and 28 from the USA) and 78 (78/82, 95%) gave consent for their information to be pooled anonymously and published (52 from the UK and 26 from the USA).

The majority of participants were male (49/78, 63%), aged 30 to 50 years old (34/78, 44%), white (38/78, 49%) and had nationality in the country where they undertook the survey (68/78, 87%) (Table 1). Most did not declare a disability (56/78, 72%). Approximately half of the respondents had completed university or college education (41/78, 53%), while a similar proportion had not (31/78, 40%) (Table 1).

**TABLE 1:** Self‐reported characteristics of participants and comparison of participants in the UK and the USA.

Question	Overall (*n* = 78)	UK (*n* = 52)	USA (*n* = 26)	*p*‐value^a^ (significance taken at *p* < 0.05)
Are you excited with what we are trying to do with the *Bionics Bus*? Yes Indifferent Not stated	77 (99%) 1 (1%)	51 (98%) 1 (2%)	26 (100%) 0 (0%)	1
Sex Male Female Not stated	49 (63%) 29 (37%) 0 (0%)	37 (71%) 15 (29%) 0 (0%)	12 (46%) 14 (54%) 0 (0%)	0.046 *
Age (years) < 18 18–29 30–50 50+	7 (9%) 11 (14%) 34 (44%) 26 (33%)	1 (2%) 9 (17%) 22 (42%) 20 (38%)	6 (23%) 2 (8%) 12 (46%) 6 (23%)	0.015 *
Disabilities* None Other Neurodivergent inc. autism and ADHD Mental health Chronic physical health	56 (72%) 3 (4%) 9 (12%) 6 (8%) 11 (14%)	33 (63%) 2 (4%) 7 (13%) 5 (10%) 10 (19%)	23 (88%) 1 (4%) 2 (8%) 1 (4%) 1 (4%)	0.031 ** 1 0.710 0.657 0.089
Ethnicity White Other Did not state	38 (49%) 18 (23%) 22 (28%)	27 (52%) 8 (15%) 17 (33%)	11 (42%) 10 (38%) 5 (19%)	0.085
Nationality British/American Non‐British/Non‐American Did not state	68 (87%) 10 (13%) 0 (0%)	48 (92%) 4 (8%) 0 (0%)	20 (77%) 6 (23%) 0 (0%)	0.075
Education level University or college graduate A‐levels, high school, or lower Not stated	41 (53%) 31 (40%) 6 (8%)	23 (44%) 24 (46%) 5 (10%)	18 (69%) 7 (27%) 1 (4%)	0.116

*Percentages in this category add to over one‐hundred since participants could have more than one disability.

^a^From Fisher's *exact test comparing the distribution of characteristics in UK vs USA participants*.

There were no country‐specific differences observed in ethnicity, nationality, or education level. The USA cohort included proportionally more female participants and a greater proportion of individuals aged under 18 and between 30 and 50 years. There was also a greater proportion of participants in the USA sample who had no self‐reported disability compared to those from the UK.

The majority of participants were *excited* about the prospect of the *Bionics Bus* (77/78, 99%). Most self‐reported feeling *confident* using day‐to‐day technology (55/78, 71%) and *enthusiastic* about new technologies (60/78, 77%). Similarly, most participants (57/78, 73%) indicated they did not struggle to find new information on technology. Almost all agreed that technology is *relevant* in healthcare (75/78, 96%) and that the *Bionics Bus* could be *useful* in delivering healthcare (55/78, 71%) and for *inspiring* young people's interest in STEM (70/78, 90%) (Table 2).

**TABLE 2:** Survey responses of participants and comparison of participants in the UK and the USA.

Question	Overall (*n* = 78)	UK (*n* = 52)	USA (*n* = 26)	*p*‐value^a^ Significance at *p* < 0.05
How do you rate yourself and your capability with day‐to‐day technology? Positive Neutral Negative	55 (71%) 20 (26%) 2 (3%)	33 (63%) 16 (31%) 2 (4%)	22 (85%) 4 (15%) 0 (0%)	0.228
How enthusiastic are you to know more about new technologies, such as artificial intelligence (AI)? Positive Neutral Negative	60 (77%) 13 (17%) 5 (6%)	40 (77%) 9 (17%) 3 (6%)	20 (77%) 4 (17%) 2 (8%)	1
Do you struggle to access new information on technologies? Yes Sometimes No Not stated	13 (17%) 2 (3%) 57 (73%) 6 (8%)	11 (21%) 2 (4%) 36 (69%) 3 (6%)	2 (8%) 0 (0%) 21 (81%) 3 (12%)	0.340
How relevant do you feel technology should be in healthcare? Positive Neutral Negative	75 (96%) 2 (3%) 1 (1%)	49 (94%) 2 (4%) 1 (2%)	26 (100%) 0 (0%) 0 (0%)	0.698
How accessible at present is Healthcare to you? (i.e., are clinics and hospitals easy to get to?) Positive Neutral Negative	29 (37%) 22 (28%) 27 (35%)	21 (40%) 18 (35%) 13 (25%)	8 (31%) 4 (15%) 14 (54%)	0.038 *
Do you get stressed attending routine health appointments? (please only answer this question if you don't have regular health check ups) Yes Sometimes No	20 (26%) 25 (32%) 27 (35%)	14 (27%) 19 (37%) 18 (35%)	6 (23%) 6 (23%) 9 (35%)	0.762
How useful do you think the *Bionics Bus* would be to deliver healthcare to you? Positive Neutral Negative	55 (71%) 17 (22%) 6 (8%)	39 (75%) 9 (17%) 4 (9%)	16 (62%) 8 (31%) 2 (8%)	0.372
Do you think the *Bionics Bus* will inspire young people to be interested in Science technology Engineering and Maths? Positive Neutral Negative	70 (90%) 8 (10%) 0 (0%)	47 (90%) 5 (10%) 0 (0%)	23 (88%) 3 (12%) 0 (0%)	1

*Percentages in this category add to over one‐hundred since participants could have more than one disability.

^a^ *From Fisher's exact test comparing the distribution of characteristics in UK vs USA participants*.

Over a third (27/78, 35%) reported that healthcare is not currently accessible to them geographically. Just over one‐quarter of participants (20/78, 26%) reported feeling stressed most of the time when attending routine health appointments; another 25 participants (32%) said they sometimes feel stressed, while 27 (35%) said they never experience any stress (Table 2).

There were no statistically significant differences between countries in participants’ self‐reported capability with technology, enthusiasm for learning about new technologies, ease of accessing new information on technologies, or perceptions of the relevance of technology in healthcare. Similarly, there were no between‐country differences in reported stress when attending routine health appointments, or in perceptions of the *Bionics Bus* as a useful means of delivering healthcare and inspiring young people's interest in STEM. In contrast, participants from the USA were significantly more likely to report that clinics and hospitals were less accessible to them geographically (*p* <0.05) (Table 2).

### 3.1 Qualitative Data Analysis

When asked about the usefulness of the *Bionics Bus*, 11 USA participants commented on its ability to enhance accessibility, noting its helpfulness for those who are ‘elderly’, who have ‘mobility issues’ and for those ‘unable to leave their homes’ (Table 3). Its benefit in terms of education was mentioned by six USA participants, including how it can help the ‘youth’, ‘school children’ and ‘post high school students’ via ‘hands‐on activities’. Six UK participants mentioned its role in education, including how it could inspire children to take part in STEM and accessibility was mentioned by five UK participants (Table 3).

**TABLE 3:** Thematic analysis data with example comments regarding the usefulness of the *Bionics bus*.

USA		UK	
Themes	Sample comments	Themes	Sample comments
Accessibility (11)	Useful for people unable to leave their homes	Education (6)	Learning about future opportunities
	They aid people who can't access medical care and make healthcare more accessible for everyone.		Inspire future children to be scientific
	Convenient for elderly with mobile issue		I like that it is there to inspire young people in STEM
	Making health care accessible to mentally challenged.		The accessibility of technology to help teach
	I think rural areas could benefit but also in town areas using it for emergency purposes.		Training for young people is essential
Education (6)	Bringing information to youth they might be aware of.	Accessibility (5)	Its ability to deliver healthcare to people who might not have access to it
	The ability to become a test subject and better understand technology via hands on activities in a fun and engaging environment.		Portable
	I think the educational part for both school children and post high school students is the most useful as they are the future of the field.		Access any ware
	The bus would be great for equipment and technology upgrades to the systems that are out with patients.		If it could be accessible to patients that struggle
Progress (2)		Positivity (5)	It looks cool
			The concept is amazing
			interesting concept

When USA participants were asked about the relevance of technology in healthcare, six noted that it should complement humans, rather than replace them. This included enabling ‘more objective decision making’. Caution was expressed that technology can fail, so it was suggested that ‘humans in healthcare know the basics and rely on their own skills before using technology’ and that ‘it should include expert clinicians’ oversight and experience’. Progress was another theme, endorsed by five, with participants mentioning how health care could be revolutionised with AI and how new technology could provide solutions to problems. Among UK participants, the role of technology in providing better care was mentioned frequently, including how it can enhance monitoring. Progress, efficiency and the ability for quicker care were each mentioned by four UK participants (Table 4).

**TABLE 4:** Thematic analysis data with example comments regarding the perceived relevance of technology in healthcare.

USA		UK	
Themes	Sample comments	Themes	Sample comments
Complementary to humans (6)	Technology enables more objective decision making. Technology can also fail, so first and foremost it's important that the humans in health care know the basics and rely on their own skills before using technology to help them.	Better care (9)	Technology advancement can help identify, prevent and cure illness The more help technology can give a patient the better Technology with only make our health and healthcare services better Enhanced monitoring
Progress (5)	Health care can be revolutionised with AI New tech allows solutions to problems Technology is very important for probing various aspects of body and disease objectively that may not be visible or accessible otherwise.	Progress (4)	Technology is very important for progress Healthcare needs to innovate times need to change and move forward. Technology is extremely important in that
Health importance (3)	Healthcare is a very critical area Every stop should be pulled out for human health	Efficiency (4)	It will improve efficiency Labour saving and diagnosis
Enhancing accessibility (2)	Advances in tech can also reduce healthcare burden by allowing at home testing and tracking.	Quicker care (4)	Too long waiting lists better care quicker care

When asked about the potential of the *Bionics Bus*, the most frequently mentioned theme, by five USA participants, was education. Participants noted its ability to ‘inspire younger generations about STEM’, particularly in places ‘that may not be able to access it or lack funds or are sceptical’. Its potential to increase accessibility was again mentioned, this time by three participants, as well as its ability to provide emergency care (by two participants) and health screening (by one participant). One participant was cautious of its use in diagnostics in the absence of clinicians to interpret data. The most frequently mentioned theme among UK participants was education, particularly in relation to teaching children and young people. Accessibility was mentioned by a similar number of UK participants, including its potential for delivering rural treatment. Healthcare improvement was commented on by five, while the ability for it to provide quicker care was mentioned by three UK participants. Finally, the potential for mental health treatment was mentioned by one participant. (Table 5)

**TABLE 5:** Thematic analysis data with example comments regarding the potential of the *Bionics bus*.

USA		UK	
Themes	Sample comments	Themes	Sample comments
Education (5)	Great way to show children in schools different pathways Educating masses about healthcare technology and especially inspiring younger generations about STEM It can bring STEM education to places that may not be able to access it or lack funds or are skeptical Spreading enthusiasm for technology in healthcare among students and families who may not typically interact with such devices closely	Education (7)	More education for school age students inspire young people to pursue STEM careers To help youngsters have an interest Brilliant teaching resource for kids In health and teaching
Accessibility (3)	Help supporting the folks who cannot get the medical easily Used as a way to possibly make healthcare more accessible.	Accessibility (7)	Easier access for people with limited mobility Rural treatment The ability to help when getting to a clinic is not an option
Emergency care (2)	Emergency response and disaster services.	Healthcare improvements (5)	Can keep people healthy Diagnosis and treatment improvements
Screening (1)	First line of medical screening,	Quicker care (3)	It can reduce waiting lists Quicker care
Caution on diagnostic potential (1)	Rather than the man it providing diagnostic / monitoring value in absence of clinical staff to interpret the collected data	Mental health (1)	Can help me with my mental health

Participants were asked to suggest ideas for the contents of the *Bionics Bus*. The most common theme among USA participants, mentioned by six, was diagnostic equipment. This included suggestions of X‐rays, non‐invasive biometric tests with real‐time results, blood pressure cuffs and electrocardiograms. Educational material was brought up by two USA participants, specifically enhancing information on artificial intelligence and to share insights into technical devices to help understand their underpinnings. Treatment planning materials, treatments and digital health reports were each mentioned by one USA participant. One of these participants mentioned the possibility of a three‐dimensional scanner for prosthetic planning, instead of needing to travel long distances for this. UK participants mostly mentioned resources relating to educational material, including information on STEM subjects. Five UK participants suggested diagnostic material, including X‐rays, blood pressure cuffs and electrocardiograms, while five endorsed any technology. Three UK participants mentioned that it could do treatments and tests instead of doctors. One UK participant mentioned that it could specifically help with cancer care (Table 6).

**TABLE 6:** Thematic analysis data with example comments regarding the suggests contents of the *Bionics bus*.

USA		UK	
Themes	Sample comments	Themes	Sample comments
Diagnostic equipment (6)	X‐ray machines	Education (8)	Information on how to get into studying STEM subjects (eg. Degree apprenticeships)
	Comprehensive biometric tests, non invasive, real time results		Accessible information
	Have the ability to do a comprehensive diagnosis in minutes. Blood pressure and EKG was good. What other noninvasive procedures can be done in a few mins.		I think the core concepts of computing need to be covered to enable the beneficiary's the gain a fundamental understanding of the technology that surrounds them.
	Basic physiological measurement devices, perhaps a simple USB ultrasound scanner, maybe a SPO2 sensor, etc		Interactive technology for learning
Educational material (2)	Info on AI, explain how to validate data	Diagnostic equipment (5)	Portable x‐ray. Ultrasound
	Opportunity to see inside some of the technical devices and how they work, interactive fun experiments, bite size knowledge takeaways, see some examples of cutting edge AI tools or BCI applications!		ECG, X‐ray, BP and other stuff
			Any diagnosis equipment for people that have difficulties accessing normal clinics.
Treatment planning material (1)	3D scanner for things like prosthetics	Technology (5)	Technology that is flexible for varied use.
			Relevant technologies for serving the health of the nation.
Treatments (1)	Lots of different first aid, medicine and all that stuff.	Instead of doctors (3)	The bus can help me have therapy which my doctor is not giving
			things which the GP does like checks
Digital health reports (1)	Have a report print out that someone can take a picture with their phone. Don't bother with paper print out because that's more waste and junk to carry.	Specific specialities (2)	Cancer care Anything mobile, heart, kidney, blood

## 4 Discussion

This study examined public attitudes toward the *Bionics Bus*, an innovative mobile STEM and digital health engagement platform, across two healthcare and educational systems: the UK and the USA. Despite marked differences in healthcare policies and socioeconomic contexts, the findings demonstrate strong cross‐national enthusiasm for the concept and broader recognition of the role of technology in shaping the future of education and healthcare. These results have implications for STEM education policy, digital inclusion initiatives and the design of public engagement strategies for emerging biomedical technologies.

Across both countries, participants expressed interest in the use of technology to improve STEM education and strong enthusiasm for learning about the *Bionic Bus* as a new digital tool that can deliver health care. This aligns with global trends showing increasing public readiness for technology‐mediated health and educational innovation. Nearly all participants viewed technology as relevant to healthcare, suggesting the growing acceptance of technologies such as artificial intelligence, robotics and digital diagnostics in routine care. These levels of enthusiasm mirror the policy ambitions of both the UK and the USA, where digital transformation is positioned as essential for sustainable health‐system modernisation. This is further strengthened by the Lord Dazi report in the UK, which described the UK health system (NHS) as being in the foothills of digital transformation, while other sectors have radically reshaped [11]. Overwhelming acceptance by participants supports that the *Bionics Bus* may be a key to the digital transformation of the healthcare systems.

It is worth noting that there was a gender imbalance in the respondents, favouring males more. This is consistent with the current troubling gender gap existing in STEM availability, access and engagement [28]. For the *Bionics Bus* to be successful, active attempts need to be made to be inclusive of addressing the gender gap. Possible solutions include proactive involvement by dedicated focus groups of girls and women to discuss what the facilitators and barriers are for engagement of STEM for females.

There was a noted lack of representation in the study population of those under 18 years of age, who are the primary target of the STEM element of the *Bionics Bus*. Similarly, most respondents were healthy adults, thus not having personal insight into health‐related needs. To address this, the *Bionics Bus* could be presented at Schools and health care settings, making it more inclusive and engaging.

Qualitative data provided further insight into how respondents conceptualise the role of technology. In the USA, participants emphasised the need for technology to augment rather than replace clinicians, reflecting the need for a cautious and optimistic approach to new technology. UK participants, meanwhile, centred their commentary on efficiency, monitoring and improved care quality, consistent with a public‐service framing of technology within the NHS, which is a universal healthcare system.

Taken together, these findings suggest a shared optimism grounded in differing motivations, which is understandable given the differences in the healthcare systems.

A notable between‐country difference emerged in perceptions of geographical accessibility to healthcare. USA participants were significantly more likely to report that clinics and hospitals were difficult to reach. This observation may reflect the vast geographical landscape and the challenges of providing healthcare using existing conventional clinic or hospital‐based systems. By contrast, the UK's NHS provides more geographically distributed primary care infrastructure, although rural and coastal communities continue to face significant inequities.

European and NHS pilots already show how mobile diagnostics can bypass long ambulance waits and allow rapid at‐home identification for a range of conditions for example, fractures, reducing emergency wait times and streamlining referrals to the appropriate hospital specialities [29]. Similarly, developing advances such as delivering physiotherapy in a hybrid manner through digital interfaces via mobile units could help reduce isolation in older adults, maintain mobility and lower the risk or severity of fall‐related injuries [30].

Perceived inaccessibility helps explain the strong emphasis among USA participants on the *Bionics Bus* as a tool for mobile diagnostics and outreach. In this context, the Bus aligns with existing American models of mobile health (mHealth), which traditionally deliver screenings, preventative care and chronic disease management to underserved regions [31]. In the UK, enthusiasm for the Bus was more often framed within the NHS's ‘Darzi Shifts’ toward community‐based care, digital readiness and prevention [11]. The Darzi report highlighted the importance of moving from hospital to community‐based care; however, how this should be done is being explored [11]. The Bionic Bus's accessibility, which was viewed as one of its strengths, can be argued to be a strong solution to this challenge. As such, the Bus resonates with both systems but for different structural reasons.

The *Bionics Bus* was widely viewed as valuable for education and for improving accessibility in both countries. Interest in educational elements, particularly hands‐on demonstrations of robotics, AI, prosthetics and diagnostic technologies, was prominent in both settings. This aligns with evidence that experiential STEM learning enhances science capital, supports career exploration and reduces psychological distance between learners and technical fields. The strong appeal to young people mentioned by participants suggests potential alignment with national STEM workforce development strategies in both the UK and the USA.

From an educational theoretical perspective, the *Bionics Bus* provides rich experiential learning opportunities, enabling learners to actively participate in the construction of knowledge rather than passively receiving information. Drawing on Kolb's Experiential Learning Theory, the *Bionics Bus* facilitates a cycle of concrete experience, reflection, conceptualisation and experimentation, key processes through which learners deepen understanding and retain new concepts [32]. The *Bionics Bus* shifts away from traditional didactic methods commonly used in primary and secondary education. It positions learners as active agents who explore, question and experiment with technologies. This learner‐centred approach promotes autonomy, intrinsic motivation and self‐directed inquiry, which are central to contemporary STEM education models.

## 5 Limitations

This study has several limitations that should be considered when interpreting the findings. First, the sample size was modest, with 78 participants included across both countries. Although sufficient for exploratory comparison, the small cohort limits statistical power and reduces the generalisability of the results to broader UK and USA populations. The USA sample had more university‐educated respondents and a better gender representation. The use of convenience sampling at public events may have introduced selection bias, as attendees at such events are likely to have pre‐existing interest in technology, engineering, or healthcare innovation. This could have inflated levels of enthusiasm for the *Bionics Bus* and technological engagement more broadly. The cross‐sectional design limits causal inference and as the responses were self‐reported, they could be subject to social desirability bias. Future longitudinal studies are recommended to minimise the above limitations.

## 6 Conclusion

The *Bionics Bus* offers more than a public‐engagement tool. It provides a comparative platform for examining how health systems, values and cultural narratives shape perceptions of biomedical innovation. The *Bionics Bus* could be the vehicle that elevates healthcare and STEM education beyond the foothills of technological advances.

## Author Contributions

**Christopher James**: conceptualisation, formal analysis, funding acquisition, investigation, project administration, supervision, validation, visualisation, writing– original draft. **Sarang Shankar**: conceptualisation, data curation, formal analysis, investigation, validation, visualisation, writing – original draft. **Madeleine Dale**: data curation, formal analysis, methodology, visualisation, writing – original draft. **Bhathika Perera**: investigation, validation, visualisation, writing – original draft. **Richard Laugharne**: conceptualisation, formal analysis, investigation, project administration, supervision, validation, visualisation, writing – review and editing. **Matthew Allen**: visualisation, writing – original draft. **Maria Feijoo‐renones**: conceptualisation, investigation, visualisation, writing – review and editing. **Rohit Shankar**: conceptualisation, data curation, formal analysis, funding acquisition, investigation, methodology, project administration, resources, supervision, validation, visualisation, writing – original draft, review and editing

## Funding

The development of this paper was enabled by the EPSRC‐funded Bionics+ Network

## Conflicts of Interest

CJ is the developer and inventor of the *Bionics Bus*, the product discussed significantly in the paper and has received research support from EPSRC. RS has received institutional and research support from LivaNova, UCB, Eisai, Veriton Pharma, Neuraxpharm, Bial, Angelini, UnEEG and Jazz/GW pharma outside the submitted work. No other author has any declared conflict of interest related to this paper.

## Supporting information




**Supporting File 1**: htl270090‐sup‐0001‐SuppMat.pdf.

## Data Availability

All data used for the paper are within the manuscript.
